# Stress-Induced Diabetes: A Review

**DOI:** 10.7759/cureus.29142

**Published:** 2022-09-13

**Authors:** Kapil Sharma, Shivani Akre, Swarupa Chakole, Mayur B Wanjari

**Affiliations:** 1 Medicine, Jawaharlal Nehru Medical College, Datta Meghe Institute of Medical Sciences, Wardha, IND; 2 Community Medicine, Jawaharlal Nehru Medical College, Datta Meghe Institute of Medical Sciences, Wardha, IND; 3 Research, Jawaharlal Nehru Medical College, Datta Meghe Institute of Medical Sciences, Wardha, IND

**Keywords:** blood sugar, diabetes mellitus type 2, hyperglycemia, insulin resistance, glucocorticoid, stress, diabetes

## Abstract

It has long been established that stress has a significant impact on metabolic function. Type 2 diabetes may be initiated by psychological and physical stress. The central and peripheral nervous systems are both involved in the neuroendocrine framework that underlies the underlying processes. The release of catecholamines and a rise in serum glucocorticoid concentrations caused by psychological stress enhance the requirement for insulin and insulin resistance. Experiencing persistent hyperglycemia in people with diabetes may be influenced by stress. Blood sugar levels may rise due to hormones being released in response to stress. Although this has adaptive significance in a healthy patient, in the long run, it can cause insulin resistance and lead to diabetes. Additionally, diabetes may cause abnormalities in the regulation of these stress hormones.

## Introduction and background

Every individual gets affected by stress, which is a human reaction. The human body is designed to be able to sense stress and react to it. Stress responses help our body acclimate to unfamiliar situations. Stress can keep us alert, inspired, and ready to avoid danger. Stress cannot be measured objectively by testing. Its presence and intensity are only discernible to the individual who is experiencing them. It frequently causes agitation, dread, exhaustion, and frustration. An individual could experience physical exhaustion, weariness, and an inability to cope. The biological reaction known as stress can be triggered by any intrinsic or external stimuli [1]. The fight-or-flight response, which includes a variety of catabolic, anti-reproductive, anti-growth, and immunosuppressive processes, is momentary and geared toward survival [2,3]. By extending their length, contributing significantly to human illnesses, and serving as a triggering or exacerbating factor, chronic stress can make these consequences harmful. Additionally, lifestyles associated with stress, sedentary life, and unhealthy diet patterns cause increased weight gain and abnormality associated with glucose and lipid catabolism [3].

## Review

Pathogenesis and causes

Stress can be acute or chronic. Both can cause a wide range of side effects, but chronic stress can have bad long-term effects on one's health. Glucocorticoids (GC) and catecholamines are the main hormonal response to stress. These hormones do not cause side effects in the acute phase but in the long run may lead to disturbed glucose homeostasis. This disturbed glucose homeostasis can lead to chronic hyperglycemia, thus leading to insulin resistance and type II diabetes [4].

Effects of glucocorticoids on the body's metabolism

**Figure 1:**
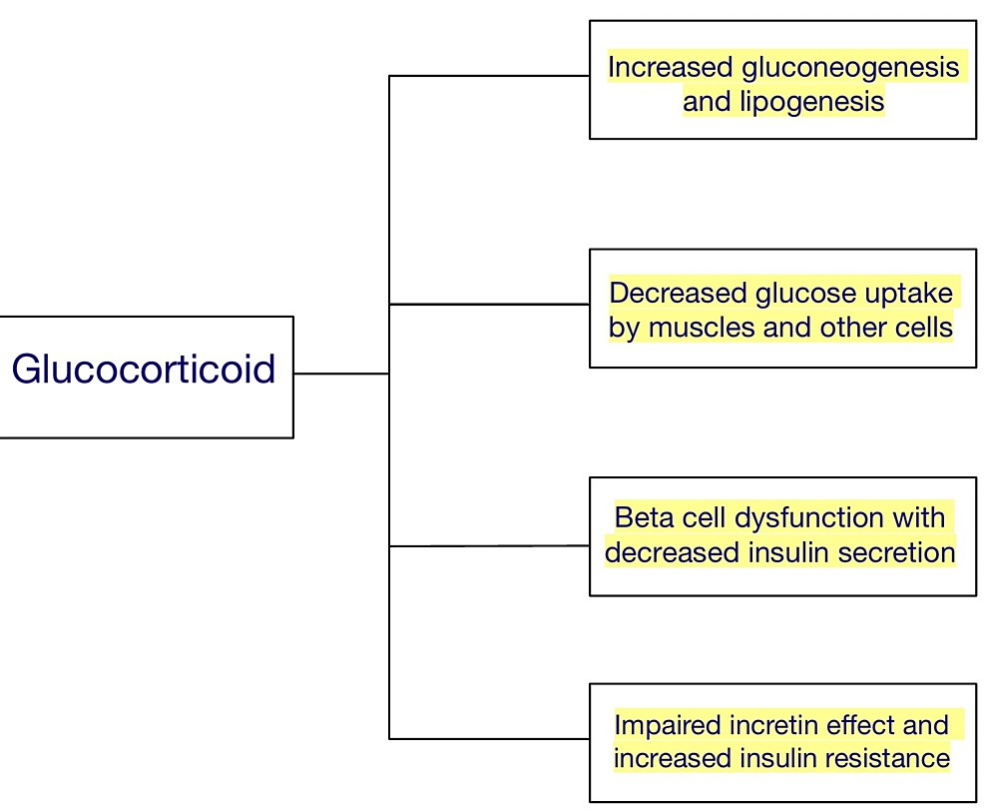
Effects of Glucocorticoids on the Body.

Glucocorticoids act by stimulating gluconeogenesis and causing the depletion of glycogen. It inhibits muscles and white adipose tissue from absorbing and using glucose, making hyperglycemia the most prevalent and quickly manifesting side effect [4]. Chronic stress can gradually cause leaner body mass loss, insulin resistance, and visceral fat accumulation. Glucocorticoids antagonize metabolic actions of insulin [5,6]. The primary regulator of glucose uptake is the glucose transporter type 4 (GLUT-4), which is seen in muscle and is stimulated by insulin. In the presence of glucocorticoids, GLUT 4 translocation to the cell surface in response to insulin is blocked, resulting in a reduction in the skeletal muscles ability to absorb glucose, causing increase in blood glucose levels [7,8]. In white adipose tissue, glucocorticoids boost lipolysis to produce glycerol, which is a precursor of gluconeogenesis, allowing the accumulation of non-esterified fatty acids within muscle cells, again causing decreased glucose uptake by interfering with insulin signaling. This leads to decreased utilization of glucose and causes a hyperglycemic state in the body. Corticosteroids also prevent cells in the pancreas from producing and secreting insulin (Figure 1) [9,10].

Effects of the sympathoadrenal system on the body's metabolism

Chronic stress can lead to increased sympathoadrenal system activity, which causes reduced glucose tolerance and an increased chance of having an acute cardiovascular event [11]. Infusing catecholamines leads to increased glycolysis, increased glycogenolysis as well an increase in gluconeogenesis, and can cause suppression of insulin-mediated glycogenesis under physiological circumstances, leading to hyperglycemia and hyperlactatemia [12]. Activating β adrenergic receptors (AR), epinephrine and norepinephrine lead to insulin resistance [13,14]. The hormone-sensitive lipase is activated by the β3 AR in adipocytes, which encourages the buildup of free fatty acids and the corresponding rise in mitogen-activated protein kinase (MAPK) activation and ceramide formation [15]. Ceramide leads to increased insulin resistance by preventing protein kinase B (PKB) from functioning [16]. As all the above processes cause increased insulin resistance in tissues, they indirectly cause the development of type II diabetes in these patients.

Type I and type II diabetes

Type I diabetes is insulin-dependent diabetes, mainly due to underlying autoimmune pathological mechanisms. It occurs mostly at a young age with sudden onset and positive serological autoantibodies test. The autoantibodies cause damage to pancreatic cells, mainly B-cells of the pancreas, leading to decreased insulin production by them causing Type I diabetes. Thus it requires insulin therapy as treatment [2-4].

In type II diabetes, the underlying cause is insulin resistance. It tends to develop in older age groups. It is mostly associated with obesity and can be reversed with lifestyle and diet modifications. In comparison to type I diabetes, Type II develops slowly over a period of time. Diet, exercise and oral medications are the main line of treatment for type II diabetes (Table 1) [5-7].

**Table 1:** Shows Difference Between Type I and Type II Diabetes

	Type I diabetes	Type II diabetes
Onset	Acute in onset	Gradual in onset
Age	Mostly seen in young age group	Seen in older age group specially with obesity
Body type	Thin or normal	Often obese
Ketoacidosis	Common	Rare
Autoantibodies	Usually present	Absent
Endogenous Insulin	Low or absent	Normal, decreased or increased

Stress and diabetes

It's indeed evident that prolonged stress and obesity create a vicious cycle that ultimately results in metabolic dysfunction. The development of insulin resistance is the result of this metabolic dysfunction. The hypothalamic-pituitary-adrenal (HPA) axis and the sympathoadrenal system have a significant role in mediating the stress response. In order for the host to survive during periods of high stress, evolutionarily preserved responses such as insulin resistance and stress hyperglycemia are produced. [17]. Stress leads to the development of stress hyperglycemia in all vertebrates, including fish, worms, and insects [17,18]. The central and peripheral neurological systems, bone marrow, white and red blood cells, and the reticuloendothelial system are among the tissues that use glucose most extensively and are non-insulin dependent cells [19]. In animal models of hemorrhagic shock, the injection of a hypertonic glucose solution increased heart function, blood pressure, and enhanced survival [20]. By promoting angiogenesis and anti-apoptotic pathways, acute hyperglycemia may prevent cell death following ischemia. Both in vitro and in vivo research have demonstrated that cardiomyocytes exposed to an insulin-free media with high glucose concentrations are resistant to pathological insults such as ischemia, hypoxia, and calcium overload, indicating that acute hyperglycemia represents an innate defense mechanism. [21]. In addition to these, stressful hyperglycemia gives the immune system and brain a source of energy at times of stress, injury, and infection. [22-24]. So from the above discussion, we can say that acute hyperglycemia during times of stress can be beneficial for the body and is a part of the evolution of the body, but when there is chronic stress in the body, this leads to the development of insulin resistance due to multiple factors including chronic hyperglycemia. Thus leading to Type II diabetes in patients with chronic stress.

Symptoms and signs of type II diabetes

Type II diabetes, also known as type II diabetes, can have a range of signs and symptoms. These include increased appetite, frequent urination, thirst, exhaustion, impaired eyesight, slow-healing wounds, recurrent infections, numbness or tingling in the hands or feet, and regions of darker skin, mainly in the armpits and neck (acanthosis nigricans). Diabetic patients have high blood glucose levels but they have a starvation-like metabolism state because the blood has plenty of glucose available but the cell cannot utilize that glucose to the development of a pathological condition known as insulin resistance in patients. So the body starts ketone body production and utilization, leading to a fruity or sweet odor from mouth, as well as positive ketone body test in urine may be present. Some modern tests like glycated hemoglobin (HbA1c) levels and ketone body tests are very helpful to diagnose patients of type II diabetes having the above symptoms.

Treatment modalities

**Figure 2:**
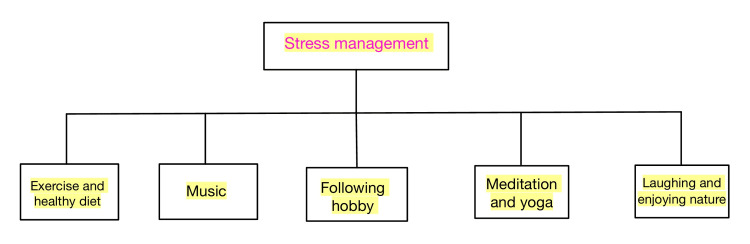
Some Ways of Stress Management.

Although you can't completely eliminate stress, you can prevent it from becoming too much by using simple daily techniques. It is very crucial for the doctor to encourage patients to get involved in good physical activities, thus decreasing stress. It has been found that physical activity has a role in stress management and also has an anxiolytic and antidepressant effect [25,26]. The patient should be encouraged to change their lifestyle and diet, which can be a significant factor for reducing stress in patients [27-29]. Patients can also follow various relaxation techniques like deep breathing, meditation, yoga, etc. Sleep constitutes a very important part of our lifestyle. It has been found that lack of sleep can lead to an increase in stress in patients [30]. Depending on the difficulties you encounter in life, stress can either be a short-term or long-term problem. You may prevent the majority of the physical, emotional, and behavioral effects of stress by regularly adopting stress management practices (Figure 2).

Management of type II diabetes

First, the patient should be encouraged to make changes in the diet and daily lifestyle routine, which can also help reduce obesity and maintain blood glucose in patients If the above modifications do not lead to significant diabetic control, in such patients oral hypoglycemic drugs should be used. The first line of drug is metformin. Metformin helps to significantly decrease the blood glucose levels to normal, it does so mainly by decreasing gluconeogenesis and increasing insulin sensitivity in tissues. Metformin decreases blood glucose without the side effect of hypoglycemia, as seen with other oral hypoglycemic drugs [31,32]. The second line of drugs in the management of type II diabetes are alpha-glucosidase inhibitors like voglibose and acarbose, which are used as adjuvants with metformin to decrease blood sugar levels. It acts by reversibly inhibiting alpha-glucosidase enzyme in the gut causing decreased and slow glucose absorption from the gut, thus helping patients by lowering their post-prandial blood glucose levels [33,34]. The third drug that can be added to this treatment is sulfonylureas. They lower blood sugar by exerting their effects on beta cells of pancreas, stimulating insulin secretion. Other oral hypoglycemic drugs include dipeptidyl peptidase-4 (DPP-4) inhibitors, thiazolidinediones (TZDs), meglitinides, etc. If oral hypoglycemic drugs fail or are contraindicated in patients, subcutaneous insulin therapy shou;d be started in such patients. Diet and lifestyle modification should be encouraged in patients, which not only decreases obesity but also helps in reversal of insulin resistance in the body. To reduce calories and fat, patients should choose meals that are higher in fiber. Focus should be on on whole grains, vegetables, and fruits [33,34].

Complications of type II diabetes

Type II diabetes is a state where there is plenty of blood glucose available, but the body is not able to utilize it due to insulin resistance in the body. In the long run, it can give rise to a number of health-related issues and can interfere with a patient's life and can also lead to severe complications. Some of the complications include nerve damage (neuropathy), renal damage (nephropathy), eye problems such as cataract and glaucoma, increased susceptibility to severe fungal infections, slow healing, hearing impairment, etc. Prevention is always better than cure, as the statement states, and patients should be encouraged to do daily activities and regular follow-ups.

## Conclusions

Numerous studies have linked potential stress, both physical and emotional,l to the emergence of type II diabetes. Stress has become a part of daily life, which causes many problems in the body's metabolism. Stress leading to hyperglycemia is an evolutionary process helping the body to go through an acute stress phase, which has been proven in several studies, especially in animals. These stress-induced changes can be very beneficial in an acute state. But when it comes to long-run effects, it can lead to various side effects on the body's way of metabolism. Chronic stress-induced hyperglycemia along with other mechanisms, causes tissue-level insulin resistance, thus leading to type II diabetes in patients with chronic stress states. This leads to various complications in the body and can be sometimes life-threatening. Preventive measures like stress management therapies should be used and encouraged. Patients should be advised to take meals with more fiber and fewer calories and fat. Lifestyle modification also plays an important role in stress as well as diabetic management. If patients fail to control blood glucose levels, they should be started on oral hypoglycemic drugs. If oral hypoglycemic drugs do not cause any significant decrease in blood glucose levels, patients should be started on subcutaneous insulin therapies. Diabetic control is very important to prevent its side effects and sometimes life-threatening complications. As a physician, it is very important to take a good history of a patient, and find if the patient has any stress history, which can be a significant underlying cause of hyperglycemia.
